# IMPROVING THE VALUE OF SERIOUS ILLNESS CARE

**DOI:** 10.1093/geroni/igad104.0027

**Published:** 2023-12-21

**Authors:** Brystana Kaufman

**Affiliations:** Duke University, Durham, North Carolina, United States

## Abstract

My Health and Aging Policy Fellowship placement with CMMI provides an opportunity to learn about the current policies impacting palliative care payment and process for developing new models. Palliative and hospice care has been one of the fasting growing health services in the U.S. and the VA over the past decade. Although Medicare’s hospice benefit requires a 6-months life expectancy, palliative care may be appropriate for seriously ill individuals depending on their clinical and psychosocial needs. Palliative care is an interdisciplinary, team-based approach that focuses on improving quality of life, aligning care with patient preferences, and relieving distressing symptoms for people with serious, life-limiting illness and their families. In practice, palliative consultations, teams and services are highly tailored to each patient and family’s needs. The heterogeneity of patient needs, care settings and services creates challenges designing value-based payment strategies. I am working on a team that will assess the feasibility of a value-based strategy for supporting seriously ill beneficiaries’ palliative care needs. Current learnings include 1) understanding the CMMI priorities as identified in their strategy refresh in 2021; 2) mapping the design elements that are relevant for palliative care across Medicare Advantage and Traditional Medicare Alternative Payment Models; 3) synthesizing evidence and leveraging my research experience evaluating value-based payment models and outcomes for high cost, high need populations in Medicare and the VA. In addition to key learnings specific to my placement, I will share how the HAP fellowship experience has impacted my research career and opportunities for the future.

